# Focused Ultrasound Ablation of an Arteriovenous Malformation Model

**DOI:** 10.3389/fneur.2021.671380

**Published:** 2021-06-03

**Authors:** Jesse Jones, Mark Bolding, Henrik Ullman, Naoki Kaneko, Satoshi Tateshima

**Affiliations:** ^1^Departments of Neurosurgery and Radiology, School of Medicine, University of Alabama at Birmingham, Birmingham, AL, United States; ^2^Department of Radiology, School of Medicine, University of Alabama at Birmingham, Birmingham, AL, United States; ^3^Department of Radiology, School of Medicine, University of California, Los Angeles, Los Angeles, CA, United States; ^4^Department of Radiological Sciences, David Geffen School of Medicine, University of California, Los Angeles, Los Angeles, CA, United States

**Keywords:** arteriovenous malformation, embolization, high intensity focus ultrasound, non-invasive ablation, MRI guided ablation

## Abstract

Brain AVMs are rare but serious vascular lesions that often pose a management dilemma between the risk of various treatment modalities and uncertain natural history during observation. We describe preliminary data on the use of focused ultrasound as a novel therapeutic strategy. In an AVM model, one session of ultrasound gradually reduced flow through the lesion without inducing rupture. Due to its non-invasive yet immediate ablative effects, focused ultrasound may allow safer treatment of AVMs. However, further studies are needed to clarify its efficacy and side effect profile.

## Introduction

The current treatment of brain arteriovenous malformations (AVM) consists of surgical resection, stereotactic radiosurgery (SRS) and/or endovascular embolization. A specific set of advantages and limitations applies to each approach or combination thereof, depending on the angioarchitecture and other patient factors (1). High intensity focused ultrasound (HIFU) is emerging as an alternative treatment for several neurological disorders including brain tumor and movement disorders (2). However, the role of HIFU in brain AVM has been less well-studied.

HIFU induces tissue ablation in a rapid, non-invasive, and non-ionizing fashion that may overcome some limitations of established brain AVM treatments by transmitting sound waves through the intact cranium onto a targeted volume of interest. Surgical resection is predicated on access to the malformation, which is untenable in deeply located lesions such as the thalamus. Location is less problematic in SRS, although ionizing radiation often leads to perilesional leukoencephalopathy and radiation necrosis. The cognitive effects of radiation in pediatric patients significantly limit its utilization. Furthermore, the year's long delay from treatment to obliterative vasculopathy prolongs hemorrhage risk, which persists until complete occlusion of the shunt. Endovascular embolization rarely produces complete AVM occlusion and rather serves a more adjunctive role to surgery and/or SRS in larger or acutely ruptured lesions.

With the limitations of conventional therapies in mind, we sought to assess whether HIFU ablation is feasible in a swine model of brain AVM.

## Methods

The study was approved by the local Animal Research Council (ARC) prior to study inception. The porcine AVM model was surgically created, as described previously (3), in a juvenile female Yucatan swine. Following a period of 4 weeks to allow maturation of the shunt, the animal was brought to the MRI suite and placed under general anesthesia. An endotracheal tube was inserted, and mechanical respiration initiated. Vital parameters, including basal temperature, respiratory rate, blood pressure, heart rate, and peripheral oxygen saturation, were continuously monitored. The animal was positioned prone with the HIFU transducer placed ventral to the mandibular angle.

**Figure 1:**
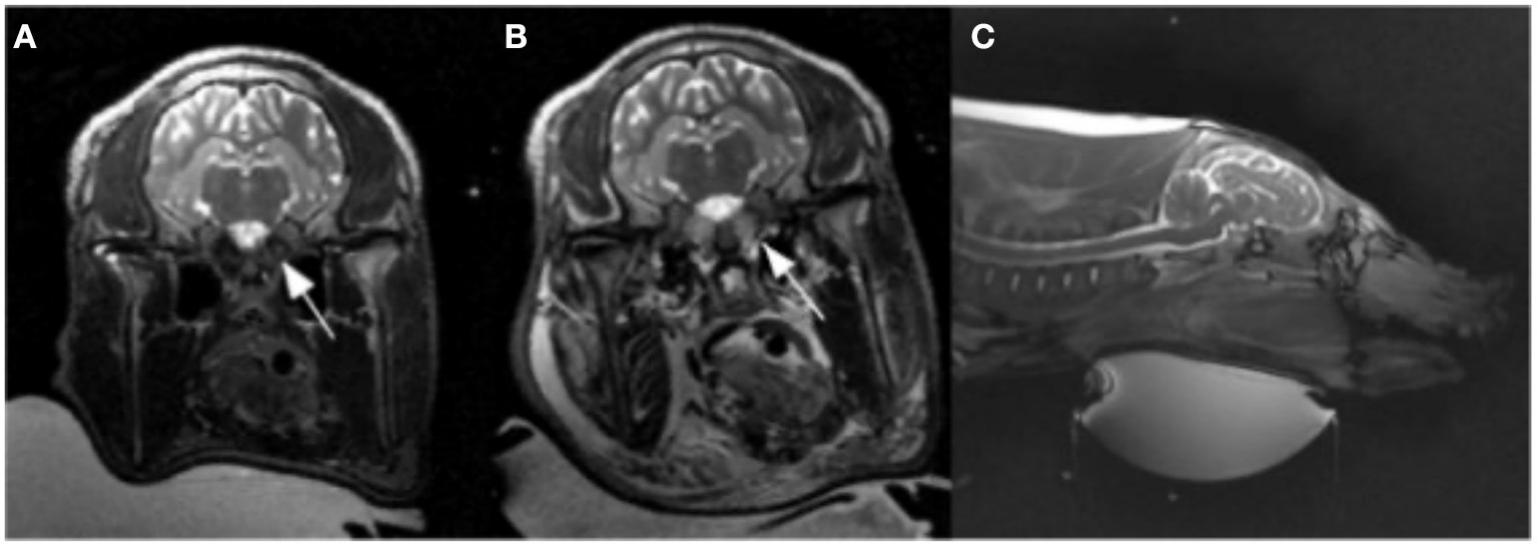
Pre **(A)** and post **(B)** ablation T2 weighted images. **(B)** Illustrates T2 prolongation within the bilateral rete mirabile (arrows) as well as the facial soft tissues, indicating edema. **(C)** T2 weighted sagittal section showing ultrasound transducer ventral approach.

Baseline MRI was performed, including T1W MPRAGE, FSE T2, and T1W contrast enhanced time-resolved MRA (CE-MRA), on a 3 Tesla MR scanner (Siemens Prisma, Erlangen Germany). CE-MRA was obtained after intravenous injection of 0.5 mmol gadobutrol (Bayer, Whippany NJ) diluted in normal saline with 1.18 s/frame and a total duration of 30.68 s. The rete mirabile was identified on anatomic sequences and high flow shunting confirmed by CE-MRA. An MR Thermographic sequence (single slice spoiled gradient echo) was initially performed through the rete, however, it was found to be non-diagnostic because of susceptibility artifact from the skull base. HIFU trajectory was planned through a ventral approach to the skull base where an acoustic window to the rete was identified (Figure 1). The bilateral rete was then targeted with a 1.0 MHz HIFU system with 128-element phased array transducer (model number LF-LAS-128, Image Guided Therapy, Pessac France) and sequentially ablated with interleaved CE-MRA to assess flow reduction. Treatment was concluded due to heating of intervening tissue (core temperature > 39°C). CE-MRA was repeated six times throughout the ablation session.

Post procedure imaging was obtained with T2 and CE-MRA sequences. At the conclusion of the study, a lethal dose of anesthetic was administered intravenously per University veterinary protocol.

## Results

**Figure 2:**
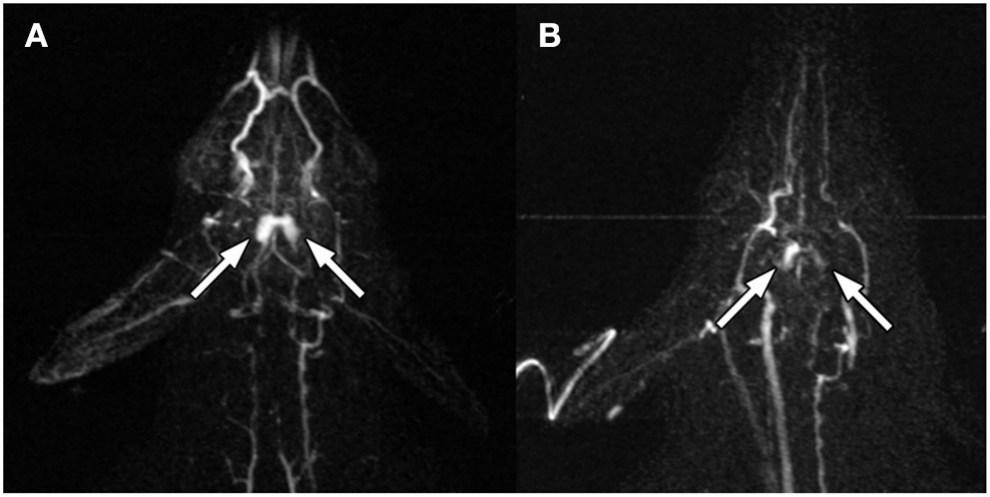
Pre- **(A)** and post- **(B)** ablation MRA at 14 s after peripheral IV gadolinium injection. The bilateral rete mirabile (arrows) show decreased contrast enhancement.

The rete mirabile was clearly visualized on pre-procedure imaging (Figure 1). CE-MRA showed T1 shortening within the nidal-type vessels of the rete, as well as rapid transit time, consistent with high vascular flow through the AVM model (Figure 2).

**Figure 3:**
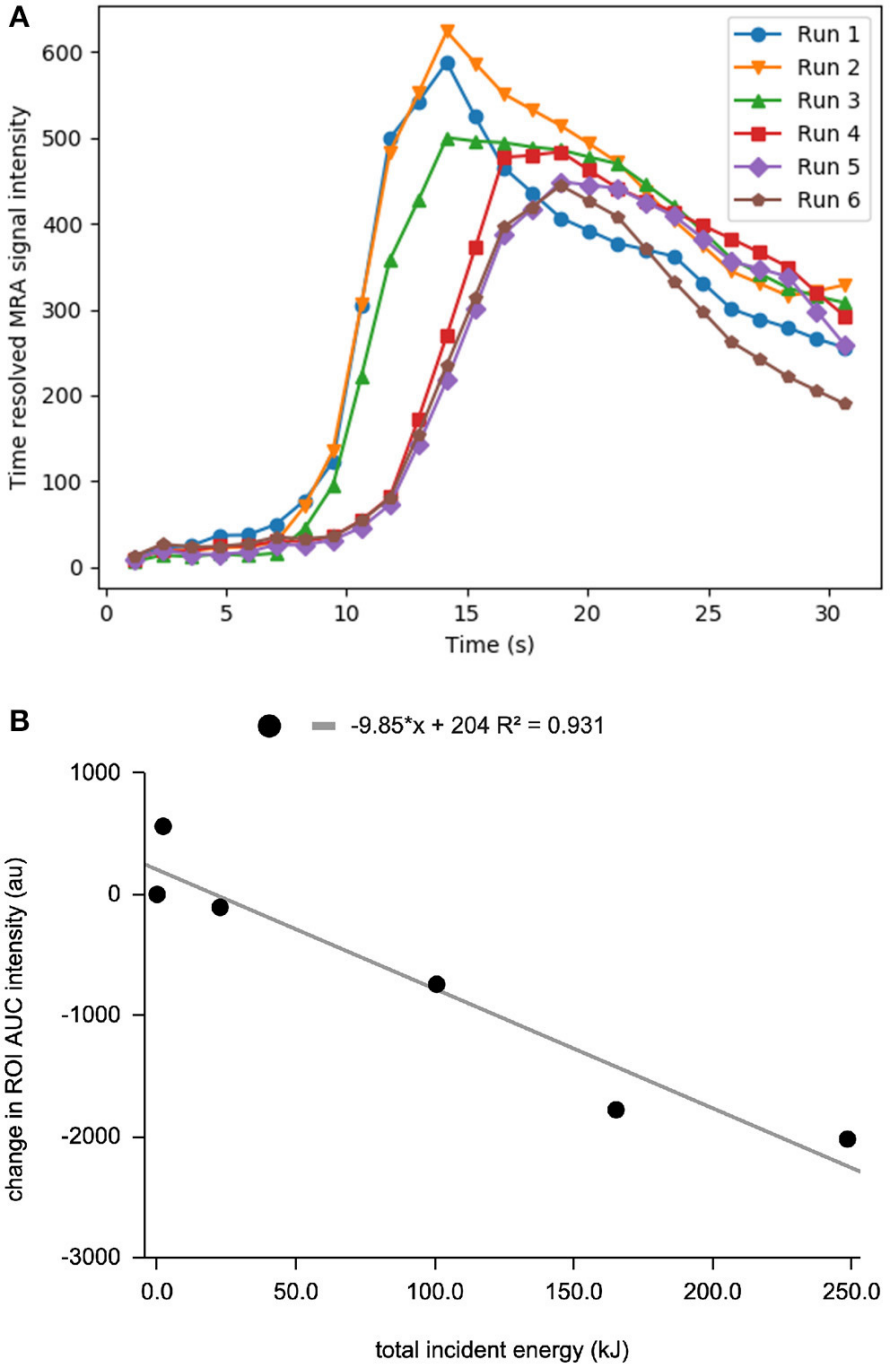
**(A)** Time resolved MRA enhancement curve of the rete mirabile at baseline and after sequential ablation sessions. Run 1 is baseline curve before treatment. **(B)** decrease in MRA intensity vs. total incident energy.

Following sequential HIFU ablation, CE-MRA showed progressive flow reduction through the rete shunt as evidenced by increasing mean transit time and time to peak, as well as decreasing blood flow (Figure 3). The final post procedure CE-MRA showed a 27% reduction in blood flow relative to baseline values and 32% reduction compared to the maximum. The area under the curve was calculated for the CE-MRA runs, showing a significant decrease in signal intensity (Figure 3B, *p* < 0.01 for Spearman *r*). Post-ablation T2 weighted MRI and necroscopy demonstrated thermal injury to the facial soft tissues along the ultrasound trajectory.

## Discussion

Despite advancements in the treatment of brain AVM over the past decades, many lesions still pose a challenge to safe and complete obliteration. Treatment related complications have in fact been shown to result in worse outcomes than the natural history, at least on short term follow-up (4). From this frustrating background also comes opportunity for novel approaches, such as HIFU.

In contrast to applications in functional neurosurgery and neuro-oncology (5, 6) HIFU for intracranial vascular lesions is less studied and no investigation of brain AVM has been published. However, data from peripheral vascular ablation by focused ultrasound in both animal models and man is encouraging (7–14).

To this background, we describe preliminary experience applying HIFU to a brain AVM model. The swine carotid rete is an established *in vivo* AVM model for SRS (15) that recapitulates the high arterial flow through a plexiform nidal structure. In the current study, we utilize this model and demonstrate its use for the development of a HIFU ablation technique. HIFU of swine AVM model is indeed feasible, as demonstrated above. Sufficient heating was achieved to markedly reduce nidus size and shunting. A workflow of sequential ablation with stepwise increasing energy, interspaced with rapid CE-MRA, allowed frequent assessment of the treatment effect.

**Figure 4:**
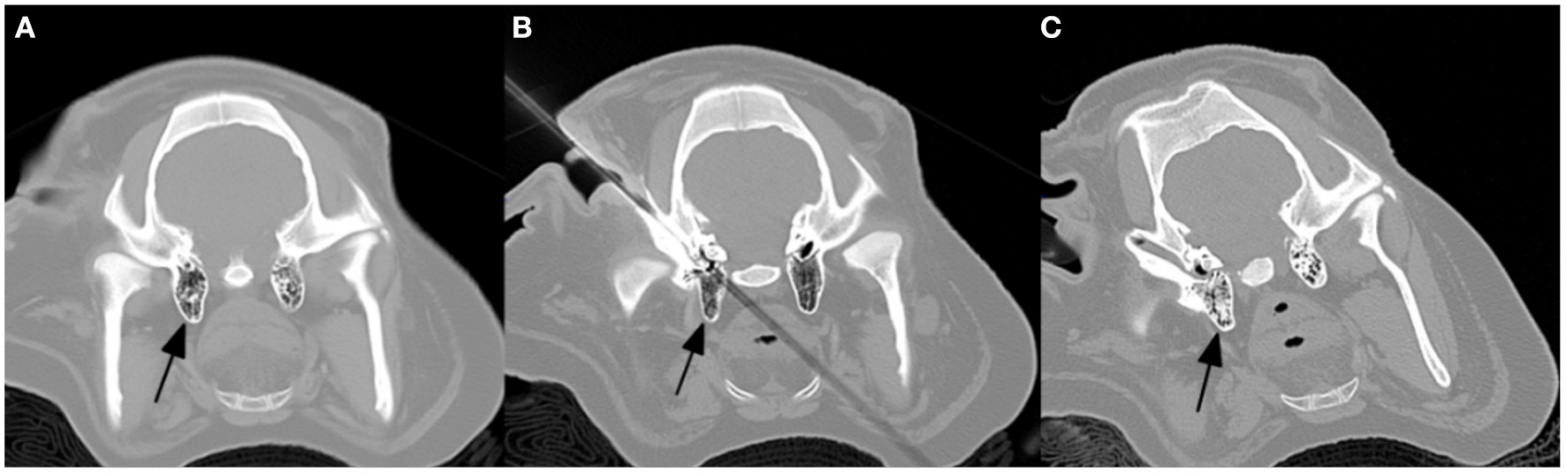
Percutaneous access of the external auditory canal and infusion of saline into the mastoid air cells (arrows) to widen the acoustic window. **(A)** pre, **(B)** percutaneous access, and **(C)** post saline infusion.

However, several challenges were evidenced by incomplete obliteration of the nidus and thermal soft tissue injury. First, the rete mirabile is difficult to insonate due to adjacent bone and air containing structures. Unfortunately, wide-array transducers currently in use for human brain HIFU applications are not amenable to the rete trajectory. We have since developed a practice of percutaneously accessing the tympanic cavities and infusing saline to widen the acoustic window (Figure 4). Additionally, MR Thermography can be utilized to monitor heat deposition along the US trajectory in real time. An US attenuation map created by returning echoes would also be helpful in estimating energy deposition at the focal spot. Second, the significant heat sink effect mediated by blood flow convection in AVM limits energy deposition. While this was partially overcome with progressively higher power, there may be a role for adjunctive flow-control techniques such as induced hypotension, balloon occlusion of feeding pedicles, or transient asystole.

Further studies are needed to continue development of HIFU for brain AVM treatment and to assess long term effects. The current swine model will aid this development, albeit with several limitations which must be improved upon. Heat deposition by the ultrasound beam could potentially result in tissue damage along the treatment path. In addition, serious complications could result if vital blood vessels are inadvertently damaged during treatment. Unlike current neurological HIFU applications (16), this model utilizes a bone window beam trajectory through soft tissue rather than cranium, which may alter ablation characteristics. Future work on this method should emphasize more refined control of the beam shape and trajectory and improved techniques for real time monitoring temperature of the tissue along the beam path and within the AVM. In conclusion, the current study provides encouraging results on the feasibility of HIFU for brain AVM. Additional preclinical work are necessary to improve ultrasonic access to the rete mirabile, assess the long term effects of treatment, and to optimize energy deposition through flow control and real time treatment monitoring strategies.

## Data Availability Statement

The raw data supporting the conclusions of this article will be made available by the authors, without undue reservation.

## Ethics Statement

The animal study was reviewed and approved by UCLA Animal Research Council.

## Author Contributions

JJ conceived the experiments, supervised the HIFU procedure, and edited the manuscript. MB analyzed the experimental data, created figures, and edited the manuscript. HU primarily wrote the manuscript. NK performed the rete anastomosis surgery and edited the manuscript. ST oversaw project design and implementation, assisted with grant funding, and edited the manuscript. All authors contributed to the article and approved the submitted version.

## Conflict of Interest

The authors declare that the research was conducted in the absence of any commercial or financial relationships that could be construed as a potential conflict of interest.

## Abbreviations

AVM: Arteriovenous malformation
CE-MRA: Contrast Enhanced Magnetic Resonance Angiography
HIFU: High Intensity Focused Ultrasound
SRS: Stereotactic Radiosurgery.
